# The Influencing Factor of MgAl_2_O_4_ on Heterogeneous Nucleation and Grain Refinement in Al Alloy Melts

**DOI:** 10.3390/ma13010231

**Published:** 2020-01-05

**Authors:** Lin Yang, Lu Wang, Mei Yang

**Affiliations:** School of Materials and Engineering, Jiangsu University of Technology, Changzhou 213001, China; yanglin@jsut.edu.cn (L.Y.); myang@jsut.edu.cn (M.Y.)

**Keywords:** aluminum, heterogeneous nucleation, grain refinement, MgAl_2_O_4_

## Abstract

Grain refinement using oxide additions is commercially feasible and ecofriendly. MgAl_2_O_4_ has a lattice structure similar to Al and small lattice misfits with Al, and it can be an effective nucleation core when it meets certain conditions. In this paper, the influencing factor of MgAl_2_O_4_ on heterogeneous nucleation and grain refinement in Al alloys was reviewed in terms of physical force, mass percent, particle size and distribution, heating temperature and duration, interface matching, lattice distortion, and chemical reactions at the liquid/solid interfaces. The existence of in situ MgAl_2_O_4_ was necessary for heterogeneous nucleation and grain refinement, and the content of MgAl_2_O_4_ was a crucial factor in grain refinement. Physical force highly enhanced heterogeneous nucleation and grain refinement through tuning of the wetting, size, and distribution of MgAl_2_O_4_ particles with little content. The heterogeneous nucleation of MgAl_2_O_4_ played a vital role in grain size reduction when the content was at a critical value. A single crystal of exogenous MgAl_2_O_4_ could also be a potent heterogeneous nucleation substrate for Al and Al–Mg alloys under a casting temperature or a high heating temperature with a short holding time for the small lattice misfits between nucleated-phase Al and the MgAl_2_O_4_ substrate, with limited lattice distortion.

## 1. Introduction

It is inevitable that oxidation occurs on the surface of Al or Al–Mg alloy melts, especially at high temperatures, due to the high affinity between oxygen and lively elements such as Mg and Al [1]. Oxide particles are generally considered to be responsible for the performance degradation of the casting; however, with preferential solidification performance, the oxides in the melt could possibly act as inoculants during the solidification process, which results in grain refinement under certain conditions [2]. An inoculation phenomenon is considered to occur when there is a decrease in the interfacial free energy between crystal nuclei and inoculants, which is believed to be the controlling factor in the inoculation process [3]. The “linear” disregistry and “planar” disregistry theories proposed by Turnbull–Vonnegut and Bramfitt can be combined to reach the minimum criterion for interfacial free energy, when crystal nuclei and inoculant lattices match each other [4,5]. The best match can be achieved under circumstances where low-index crystallographic planes and directions of crystal nuclei and inoculants are parallel and the lattice parameters in these planes are either similar or integral–multiple.

Because MgAl_2_O_4_ has a similar crystal structure to and nearly twice the multiple lattice parameters as aluminum, in addition to a natural formation tendency on the Al alloy surface [6], it is more likely to form low-energy interfaces between Al and MgAl_2_O_4_ in their cube-on-cube orientations [7]. MgAl_2_O_4_ is a potentially vital nucleation agent for Al alloys, and closer attention has been paid to endogenous (in situ) and exogenous MgAl_2_O_4_ oxides in terms of their nucleation and grain refinement effectiveness. Endogenous (in situ) oxides can be formed through in situ reactions through the addition of oxide particles or oxygen sources external to the melt, which are usually under some physical fields and mechanical forces during the solidification. For example, in situ MgAl_2_O_4_ particles can be formed through the addition of various oxides or acids, such as SiO_2_ [6], TiO_2_, and H_3_BO_3_ [7], upon the reduction of oxides and the consumption of Al and Mg in the matrix alloy. They can also occur through oxygen introduced by a ceramic tube into the Al–Mg melts above the liquid temperature. Unfortunately, endogenous MgAl_2_O_4_ oxides that naturally form in the Al–Mg alloys always adsorb onto the surface of the alloy melt in the form of liquid-like films or clusters consisting of numerous individual micron-sized particles, which make it difficult to initiate heterogeneous nucleation [1]. As a result, physical fields and mechanical forces (including mechanical stirring using impeller-intensive shearing in a special unit with a 500-rpm screw rotation speed) and ultrasonic cavitation have been introduced into the alloy melt in order to break films or clusters into fine pieces, which can improve the heterogeneous nucleation potency of MgAl_2_O_4_ [8,9]. Li et al. [10] investigated the heterogeneous nucleation effect of MgAl_2_O_4_ particles in Al–Mg alloys with different Mg contents through intensive melt-shearing treatment and provided MgAl_2_O_4_, which acted as a potent site for the nucleation of *α*-Al grains from their cube-on-cube orientation relationship (OR). Wang et al. [2] and Kim [11] studied the characterization of MgAl_2_O_4_ formed in Al–Mg alloys with mechanical stirring or different oxidation times at 750 °C and discussed the nucleation possibility of endogenous MgAl_2_O_4_ particles acting as a nucleated substrate in terms of their lattice mismatch. Cube-on-cube ORs, {111} <110>Al||{111} <110>MgAl_2_O_4_, and {010} <001>Al||{010} <001>MgAl_2_O_4_ were experimentally observed while under continuous stirring. The lattice misfits of the two ORs were both calculated to be ~0.19%, which proved the potent nucleation ability of MgAl_2_O_4_ for Al. Recently, ultrasonic treatment (UT) and ultrasonic cavitation were found to facilitate the deagglomeration and disintegration of MgAl_2_O_4_ clusters into fine particles and nanoparticles, providing bulk nucleation sites for Al in Al–Mg alloys. An in situ Al–MgAl_2_O_4_ master alloy was synthesized through the reaction of an oxide precursor or through H_3_BO_3_ acid addition assisted by ultrasonic treatment. The grain refinement effects of in situ MgAl_2_O_4_ particles on Al and Al–Mg alloys were researched by R.S. Harini et al. [12,13] and R. Raghu et al. [7]. It was found that Al and Al–Mg alloys benefited from a 7–8-fold and even up to an 11–12-fold reduction in grain size due to ultrasonic cavitation and acoustic streaming because they provided wetting and the uniform dispersion of MgAl_2_O_4_ particles for nucleation. R. Haghayeghi and M. Qian [14] observed (in situ) a near-atomic resolution nucleation process in a liquid Al–10Mg (wt %) alloy with in situ MgAl_2_O_4_ seeds dispersed into the melt through ultrasonic vibration as a nucleated agent from a superheated temperature to approximately the liquid temperature of the alloy. The nucleation process started from three ordered layers of Al atoms that formed and remelted alternately at a superheat of 73 °C to three similar and more stable ordered layers of Al atoms formed at an approximate liquid temperature of 607 °C, which entailed subsequent crystallization.

The cube-on-cube OR of {010} <001>Al||{010} <001>MgAl_2_O_4_ was also observed in the heterogeneous nucleation of pure Al on a complete or part-MgAl_2_O_4_ reaction layer with a different morphology and thickness formed at a different heating temperature between the Al and MgO single-crystal substrate [15,16]. Small nucleation undercooling, good cube-on-cube ORs, an estimated negative Gibbs free energy for the reaction, and a calculated low lattice misfit all indicated that the new MgAl_2_O_4_ reaction product acted as a new nucleated agent instead of the original MgO substrate.

The heterogeneous nucleation effect of exogenous MgAl_2_O_4_ oxides acting as direct nucleated substrates has been investigated at a high heating temperature in Al/MgAl_2_O_4_ systems by Zhang et al. [17]. It was noted that there was an Al_2_O_3_ dendritic reaction from the Al melt and MgAl_2_O_4_ substrate, and meanwhile, part of the MgAl_2_O_4_ substrate (which had a new crystal plane) was exposed to the Al melt. Differently from the above cube-on-cube ORs, a new OR between Al and freshly exposed MgAl_2_O_4_ substrate was observed ({111} <011>Al||{200} <013>MgAl_2_O_4_ with a large misfit of about 8.36%), as was a distorted layer that was about 1 nm thick that relieved the strain between the matching planes. Both the microreaction layer and the bulk nucleated substrate of MgAl_2_O_4_ were exogenous oxides that were retained outside of the Al melt during the nucleation process.

Though various industrial grain refiners that have been discovered have been successfully used to refine aluminum, including Al–Ti–B, Al–Ti–C master alloys, and even some novel refiners with significant grain refinement effects, the release of fluoride and chloride gases and the formation of slag for the preparation of master alloys have serious adverse effects on the environment. Therefore, research to find a suitable nucleated agent and grain refiner for Al and Al–Mg alloys is still necessary [18]. Exploration of the use of in situ or exogenous MgAl_2_O_4_ particles as heterogeneous nucleating sites for Al and Al–Mg alloys is a broad task, and it has attracted more interest since MgAl_2_O_4_ particles themselves are commercially viable and environmentally friendly. The present study explores and summarizes the influencing factors of MgAl_2_O_4_ in heterogeneous nucleation and grain refinement on Al alloy melts.

## 2. Interactions between Melt and MgAl_2_O_4_ Particles

### 2.1. In Situ MgAl_2_O_4_ Particles

The similar crystal structure between Al and MgAl_2_O_4_ provides an opportunity to form a low-energy interface between them and any of their orientations. Meanwhile, MgAl_2_O_4_ displays nice wettability with Al alloys, and there are no chemical reactions between Al and MgAl_2_O_4_ at low temperatures. All of these characteristics probably make MgAl_2_O_4_ particles act as potential heterogeneous nucleation sites and further enhance the grain refinement of alloys. MgAl_2_O_4_ particles can be synthesized in situ in Al–Mg melts through the addition of oxides or other oxygen sources, such as SiO_2_, TiO_2_, B_2_O_3_, and H_3_BO_3_ [7,19,20]. Negative Gibbs free energy in the reactions between oxides and Al–Mg melts at processing temperatures of 650–900 °C suggests that the formation of an MgAl_2_O_4_ phase is thermodynamically stable. However, it is difficult to obtain a complete reaction of parent oxides and to disperse the in situ MgAl_2_O_4_ particles into the alloy melt, thus leading to the poor nucleation ability of MgAl_2_O_4_. In order to resolve this question, physical fields such as mechanical stirring, ultrasonic treatment, and intensive melt shearing have been introduced, which has resulted in in situ MgAl_2_O_4_ forming with, on average, small particle sizes and a diffuse distribution in the melt.

Using ultrasonic treatment, one reaction resulted in the massive growth of MgAl_2_O_4_ particles from an Al–Mg melt, and the oxides could be attributed to the constant removal of MgAl_2_O_4_ particles on the oxides’ surface and to the pushing of MgAl_2_O_4_ particles into the melt, thus exposing fresh oxide surfaces and allowing for the advancement of the bulk oxide reaction [13]. Ultrasonic treatment is used as an effective physical tool to enhance the heterogeneous nucleation of an Al–Mg melt from three fields. Firstly, ultrasonic treatment introduces ultrasonic cavitation, producing intense local hotspots of temperature (5000 °C), high pressure (100 MPa), and microjets (100 m/s) [21]. A high local temperature and pressure assist with the reaction of Al and Mg atoms on the surfaces of oxides, leading to the in situ formation of MgAl_2_O_4_ crystals. Secondly, microjets aid in the fragmentation of oxides as well as in the removal of MgAl_2_O_4_ crystals, exposing fresh oxide surfaces for further reactions. Lastly, acoustic streaming and ultrasonic treatment aid in the dispersion of small MgAl_2_O_4_ crystals, which are bonded by a van der Waals force. These in situ MgAl_2_O_4_ crystals grow with low index faces, such as {220}, {311}, and {400}, and have low interfacial energy with Al [12,13]. 

The experimental parameters, including the melting and solidification methods, the alloy compositions and melt treatments, the compositions of the Al–MgAl_2_O_4_ master, the addition contents and modes of the master, and the ultrasonic treatment processes, can affect the size of the microstructural components. Therefore, studies with similar experimental methods and parameters were selected to assess the grain refinement effects of MgAl_2_O_4_ on Al alloys. The refinement degrees of the average alloy grain sizes in References [7,12,13,18] (with different contents of MgAl_2_O_4_ and different ultrasonic treatment (UT) processes) are summarized in Table 1. 

In the table, it can be seen that a significant grain size reduction was observed in pure Al and Al–Mg alloys with in situ MgAl_2_O_4_ and ultrasonic treatment, while few grain refinements (a reduction of 2–3-fold) were obtained with just in situ MgAl_2_O_4_ and hardly any grain refinements were obtained (a reduction of ~1.1-fold) with just ultrasonic treatment. This implies that the combination of in situ MgAl_2_O_4_ and ultrasonic treatment contributes to a significant grain size reduction in alloys. The presence of MgAl_2_O_4_ particles acting as nucleated sites is critical, and ultrasonic treatment results in the in-situ-formed MgAl_2_O_4_ being nanoscale-sized, with a uniform dispersion and a 2–4-fold increased number density when compared to previous formulations. The mean grain size of MgAl_2_O_4_ was almost equivalent with different UT times and temperatures. It is worth noting that the grain refinement effects with the above melting and solidification methods, UT temperatures and times, and mean grain sizes of MgAl_2_O_4_ in the different experiments were similar except for the larger grain refinement effect obtained with a short UT time (30 s) with pure Al compared to more UT time (5 min) with the Al–Mg alloy. This suggests that UT time and temperature do not play decisive roles in grain refinement. The same refinement degree for average alloy grain sizes of the Al–4Mg alloy with different mass percents of MgAl_2_O_4_ (0.58 wt % and 3 wt %) was achieved due to the different lengths of the samples (Φ 20 × 80 mm and Φ 20 × 120 mm ) in the experiment. Therefore, the effect of the mass percent of MgAl_2_O_4_ on grain refinement was then mainly studied and discussed in terms of another physical force, intensive melt shearing.

**Figure 1:**
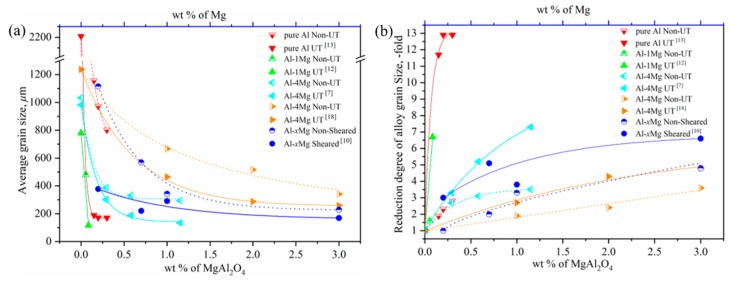
(**a**) The average grain size with respect to the mass percent of MgAl_2_O_4_ or Mg with UT or an intensive melt shearing process; (**b**) the reduction degree of the grain size relative to the mass percent of MgAl_2_O_4_ or Mg with UT or an intensive melt shearing process, with data from References [7,10,12,13,18].

Intensive melt shearing is another approach used in grain refinement treatments of Al and Al–Mg alloys. When given enough oxidation time, porous MgO initially forms on the surface of the Al–4Mg alloy, and MgAl_2_O_4_ particles later form, covered with a thin layer composed of Al_2_O_3_ from the reaction of liquid aluminum and oxygen, which is introduced from the air through the porous MgO. Therefore, it seems that MgAl_2_O_4_ generated naturally during oxidation has difficulty serving as a direct substrate for the nucleation of Al due to the separation of Al_2_O_3_ [11]. Due to the similar crystal structure of Al and MgAl_2_O_4_ and due to the MgAl_2_O_4_ solid phase forming in the melt prior to the solidification of Al, it has been proven that intensive melt shearing breaks up the MgAl_2_O_4_ oxide films and disperses the potent oxide particles, which leads to potent heterogeneous nucleation and a grain refinement effect in Al–Mg alloys. Detailed research has been carried out on the effect of the Mg content and intensive melt shearing on grain refinement in Al–Mg alloys [10]. Al–Mg alloys were prepared in an electric resistance furnace using commercial purity Al and Mg. After Mg was dissolved under the protection of Ar gas, the melt was held at a constant temperature of 700 °C for 4 h to assist in the natural reaction of MgAl_2_O_4_, with an average grain size of hundreds of nanoscales. The sheared samples were prepared through sheared melting for 60 s at 700 °C and then poured into a copper mold at a consistent cooling rate of 3.5 K s^−1^ in the central region. The dimensions of the mold were about 25 mm in diameter at the bottom, 60 mm in diameter at the top, and 65 mm in length. The nonsheared samples were treated in the same way without the melting and shearing. Significant grain refinement was achieved with intensive melt shearing or by increasing the content of Mg when it was less than 1 wt %. When comparing the grain sizes, there was a critical Mg content observed, around 2 wt %, regardless of whether the Al–Mg alloys sheared or not, as they were nearly equivalent and almost constant. A similar result was observed in the Al–5Mg alloy with the addition of Ti. Intensive melt shearing introduced further grain refinement when the content of Ti was less than 0.05 wt %, while the grain sizes of the nonsheared and sheared Al–5Mg alloys were constant with more than a critical content of 0.05 wt %. The effect of the mass percent of MgAl_2_O_4_ or Mg with UT or an intensive melt shearing process on grain refinement is comprehensively considered and illustrated in Figure 1 with data from References [7,10,12,13,18].

It is interesting to note that in Figure 1a, the average grain sizes of the pure Al and Al–Mg alloys obviously decrease with an increase in the content of MgAl_2_O_4_ or Mg, which is in the low range of 0 to 1 wt %. At the same time, UT and intensive melt shearing further highly strengthen the grain size reduction in the low content range of MgAl_2_O_4_ or Mg, which suggests that physical force is an effective method in breaking up the MgAl_2_O_4_ film into nanoparticles, dispersing MgAl_2_O_4_ particles into melts, and then enhancing the heterogeneous nucleation effect of MgAl_2_O_4_ particles. However, with a continuing increase in the content of MgAl_2_O_4_ or Mg, the average grain size decreases slowly and has a gradual leveling tendency: meanwhile, the effect of the size reduction is small under physical force when the content of MgAl_2_O_4_ or Mg is more than the critical value of around 2 wt %. In order to understand the respective contributions of endogenous MgAl_2_O_4_ particles and the physical force of UT or shearing, the reduction degree of grain size with respect to the mass percent of MgAl_2_O_4_ or Mg with UT or a shearing process was converted into Figure 1b from Figure 1a. It can be seen that the contribution of endogenous MgAl_2_O_4_ to grain size reduction is 2–3-fold, while that of physical force is about 5–8 and up to 11–12-fold when the content of MgAl_2_O_4_ or Mg is in the low range, less than 1 wt %. The contribution of physical force to grain size reduction becomes stable after reaching the critical content of MgAl_2_O_4_ or Mg.

Combining the above experimental results and analysis, it is clear that physical force plays a more important role when there is an assured existence of endogenous MgAl_2_O_4_ in a low content range. This implies that physical force enhances the heterogeneous nucleation and grain refinement of Al by tuning the wetting and distribution of MgAl_2_O_4_ particles. In addition, and more importantly, it is clear that the MgAl_2_O_4_ content is a critical factor in the grain refinement of Al and Al–Mg alloys, since similar grain sizes are obtained with or without physical force when the content of MgAl_2_O_4_ or Mg exceeds 2 wt %. This also proves that the endogenous MgAl_2_O_4_ phase is a potent heterogeneous core for the nucleation of Al and Al–Mg alloys and plays a vital role in grain size reduction under the following conditions: a uniform distribution, hundreds of nanoscale-sized to several microscale-sized particles, and a mass percent of about 2 wt % (whether there is physical force or not).

### 2.2. Exogenous MgAl_2_O_4_

Both MgO and MgAl_2_O_4_ are common oxides formed during the preparation and remelting processes of Al–Mg alloys and are regarded as being effective heterogeneous nucleating agents for Al-based alloys due to their similar lattice structures and small lattice misfits. However, some researchers have reported that liquid Al reacts with MgO substrates in various exposed crystal planes over a wide temperature span [16,17,22,23]. In these research results, Sun [16] found MgAl_2_O_4_ is a final product at a normal casting temperature between 700 °C and 800 °C with a holding time of 3 min. MgAl_2_O_4_ products are straight, and distinct layers or small islands with thicknesses of 10–40 nm form between Al and MgO. This can be referred to as exogenous MgAl_2_O_4_ in this case due to the reaction product MgAl_2_O_4_ always being retained outside of the Al melt during the nucleation process. The cube-on-cube ORs between Al, MgAl_2_O_4_, and MgO have also been confirmed by Sun, such as {200} <001>Al||{200} <001>MgAl_2_O_4_||{200} <001>MgO, {220} <001>Al||{220} <001>MgAl_2_O_4_||{220} <001>MgO, and {111} <110>Al||{111} <110> MgAl_2_O_4_||{111} <110>MgO. Both the cube-on-cube ORs and small nucleated undercooling (3–8 °C) suggest that MgAl_2_O_4_ is a perfect catalyzer for the nucleation of liquid Al.

**Figure 2:**
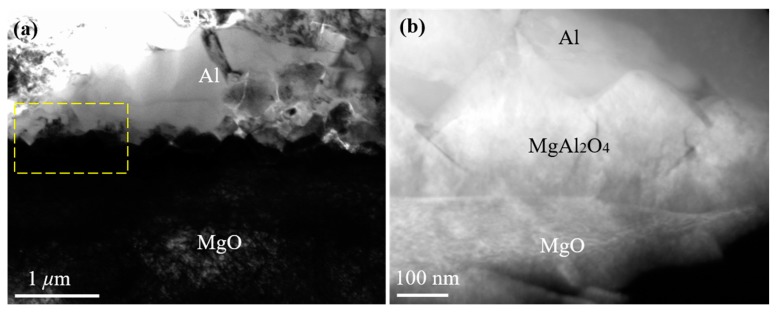
(**a**) A typical cross-sectional transmission electron microscope (TEM) image of the Al/ {001} MgO interface. (**b**) A high-angle annular dark-field (HAADF) Z-contrast image of the Al/MgO interface, which corresponds to the frame of (a).

J. Morgile et al. [22,23] have clarified that MgAl_2_O_4_ is an intermediate product and Al_2_O_3_ is a final product of the reaction at a high temperature between the Al melt and MgO substrate. Similarly to Morgile’s results, the reaction product MgAl_2_O_4_ (with a thickness between 100 and 300 nm) was also seen at the Al/MgO interface in our research. High-purity Al (>99.999%) was melted at 1027 °C with a holding time of 5–10 s and then cooled on the selected terminated planes of the MgO substrate from 1027 °C, with a cooling rate of 20 K/s in a high-vacuum chamber. The interface between MgAl_2_O_4_ and MgO was approximately straight, while the interface between MgAl_2_O_4_ and Al was a zigzag, as seen in Figure 2. To further confirm the morphology of MgAl_2_O_4_, a high-angle annular dark-field (HAADF) Z-contrast image of the Al/MgO interface was obtained (Figure 2b, corresponding to the frame of Figure 2a). Because the image contrast is proportional to the atomic mass, the contrast of the Al area is bright, while that of the MgO area is dark. The contrast of MgAl_2_O_4_ is distinct from Al and MgO, with a straight interface with MgO and a zigzag interface with Al.

The morphology and thickness of MgAl_2_O_4_ at the Al/MgO interface were different from the straight and distinct layer or small islands of MgAl_2_O_4_ formed at a normal casting temperature (between 700 and 800 °C). This suggests that the different reactions of Al and MgO occur at different heating temperatures, leading to a different morphology and thickness of the MgAl_2_O_4_ product. It can be concluded that the exogenous MgAl_2_O_4_ layer formed at the interface of pure Al and MgO can act as a new heterogeneous nucleation substrate for Al when the heating temperature is at a normal casting temperature or at a higher temperature with a holding time of a few seconds.

The heterogeneous nucleation effect of exogenous MgAl_2_O_4_ oxides acting as direct nucleated substrates was investigated at a high heating temperature by Zhang et al. [17] in an Al/MgAl_2_O_4_ system at 1027 °C with a holding time of 30 s. An Al_2_O_3_ dendritic reaction from the Al melt and MgAl_2_O_4_ substrate was noted, and meanwhile, part of the MgAl_2_O_4_ substrate (with a new crystal plane) was exposed to the Al melt. As a result, differently from the above three cube-on-cube ORs, the OR between the Al and freshly exposed MgAl_2_O_4_ substrate was {111} <011>Al||{200} <013>MgAl_2_O_4_ with a large misfit of about 8.36% and a distorted layer about 1 nm thick, which relieved the strain between the matching planes. This indicates that MgAl_2_O_4_ substrates easily form alumina and are not potent nucleation substrates for Al alloys at a high heating temperature and long holding time.

**Figure 3:**
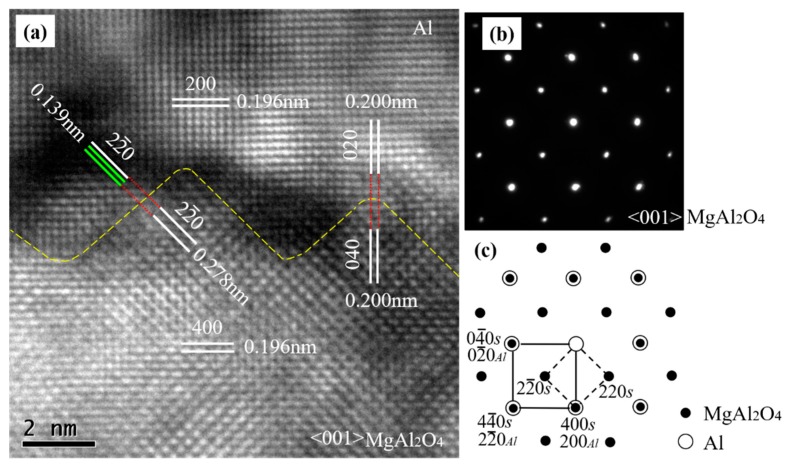
(**a**) High-resolution TEM image of Al/MgAl_2_O_4_ taken in the <001> MgAl_2_O_4_-zone direction; (**b**) selected area electron diffraction (SAED) pattern taken from both the MgAl_2_O_4_ (spinel, S) particle and the adjacent Al; and (**c**) the schematic of the pattern indexed along the <001> axis for both the MgAl_2_O_4_ and Al crystals, showing the cube-on-cube crystallographic orientation relationship (OR) between the two phases.

To understand the direct effect of the exogenous MgAl_2_O_4_ substrate on the heterogeneous nucleation of Al at 1027 °C with a short holding time of 3–5 s, high-resolution transmission electron microscopy (TEM) was carried out at the Al/MgAl_2_O_4_ nucleation interface in the <001> MgAl_2_O_4_-zone direction. Figure 3b is a selected area electron diffraction (SAED) pattern taken from both MgAl_2_O_4_ and the adjacent Al matrix along their <001> zone axes across the MgAl_2_O_4_/Al interface in Figure 3a. The SAED pattern on the MgAl_2_O_4_/Al interface shows overlapped spots. Through an analysis of the SAED pattern, we found two sets of diffraction patterns. One set was a face-centered cubic (FCC) structure with a d-spacing of 0.196 nm and 0.278 nm, which corresponded to {400} and {2$\bar{2}$0} planes of MgAl_2_O_4_ (as indexed with small solid circles in Figure 3c). The other set was also an FCC structure, with d-spacing of about 0.196 nm and 0.139 nm, which corresponded to {200} and {2$\bar{2}$0} planes of Al (as indexed with large open circles). Figure 3c gives a schematic indexing of the SAED pattern, indicating that the same crystal planes and the same crystal directions were parallel to each other in the MgAl_2_O_4_ and Al crystals. From the evidence of the SAED, it is clear that there was a cube-on-cube OR between the MgAl_2_O_4_ and Al matrices, which was {200} <001>Al||{400} <001>MgAl_2_O_4_.

It is worth noting that both MgAl_2_O_4_ and Al had an FCC crystal structure and that the lattice parameter for MgAl_2_O_4_ was 0.80831 nm, about double that of Al (0.40494 nm) [2]. The theoretical crystal plane spacings of {400} MgAl_2_O_4_ and {200} Al were 0.2021 nm and 0.2025 nm, respectively. Theoretically, the lattice misfits for the parallel crystal planes between Al and MgAl_2_O_4_ in Figure 3a were small, and the calculation results can be seen in Table 2.

The calculated lattice misfits of the different parallel planes between Al and MgAl_2_O_4_ were near those (0.09%) of the Al and Al_3_Ti monolayer between the Al and TiB_2_ substrate [24], suggesting that MgAl_2_O_4_ acts as a potent heterogeneous substrate that can be considered for actual industrial applications. Actually, well-defined atomic rows and lattice planes can be identified in Figure 3a. As can be seen in Figure 3, the {200} Al plane was parallel to the {400} MgAl_2_O_4_ plane, and the plane spacing of {200} Al was the same as that of {400} MgAl_2_O_4_. Meanwhile, {020} Al was parallel to {040} MgAl_2_O_4_, and their d-spacings were also equal to each other. The {2$\bar{2}$0} Al was parallel to the {2$\bar{2}$0} MgAl_2_O_4_, while the *d*-spacing (0.278 nm) of the {2$\bar{2}$0} MgAl_2_O_4_ was double that of (0.139 nm) {2$\bar{2}$0} Al. The parallel crystal planes, the crystal directions, and the same or integral multiple-crystal-plane spacing indicated that the cube-on-cube OR {200} <001>Al||{400} <001>MgAl_2_O_4_ was the expected value.

According to theoretical lattice misfit calculations, there should be small lattice misfits in Al/MgAl_2_O_4_ along three sets of parallel crystal planes. However, the crystal plane spacings between the three sets of parallel crystal planes were the same or double. This means that there was lattice distortion at the interface, which was consistent with our previous study, where the nucleated-phase Al fit the substrate with limited lattice distortion within a small lattice misfit (*f* < 3.1%) [25]. These studies prove that both in situ MgAl_2_O_4_ (using physical force) and endogenous MgAl_2_O_4_ (controlling for the heating temperature) are possibly potent heterogeneous nucleation substrates in the heterogeneous nucleation of Al alloys.

## 3. Conclusions

Both in situ MgAl_2_O_4_ (using a physical tool in the melt) and the exogenous MgAl_2_O_4_ crystal (controlling for the heating temperature and holding time outside of the melt) are possible candidates for use as potent heterogeneous nucleation substrates for Al alloys because of their similar crystal structures and small lattice misfits.

1. The existence of in situ MgAl_2_O_4_ means a potent heterogeneous core, and it is the premise for heterogeneous nucleation and grain refinement in Al and Al–Mg alloys. Physical force highly enhances heterogeneous nucleation and grain refinement through tuning of the wetting, size, and distribution of MgAl_2_O_4_ particles. The content of MgAl_2_O_4_ is a crucial factor in grain refinement. The heterogeneous nucleation of MgAl_2_O_4_ plays a vital role in grain size reduction when the content of MgAl_2_O_4_ arrives at a critical value. The optimal addition of in situ MgAl_2_O_4_ has the following conditions: a uniform distribution, hundreds of nanoscale-sized to several microscale-sized particles, and a mass percent of about 2 wt %.
2. MgAl_2_O_4_ reactions (from Al and MgO substrates) are retained outside the Al melt during the nucleation process and can act as potent nucleation substrates for Al alloys. Different heating temperatures control the extent of the reaction of the Al and MgO and lead to different morphologies and thicknesses of the MgAl_2_O_4_ product.
3. The exogenous MgAl_2_O_4_ single crystal is also a potent heterogeneous nucleation substrate for Al and Al–Mg alloys under a casting temperature or a high heating temperature with a short holding time. The cube-on-cube OR of {200} <001>Al||{400} <001>MgAl_2_O_4_and the same or integral multiple-crystal-plane spacing indicate that nucleated-phase Al fits the substrate with limited lattice distortion for small lattice misfits between Al and MgAl_2_O_4_.

## Figures and Tables

**Table 1:** The refinement degree of alloy grain sizes with different contents of MgAl_2_O_4_ and different ultrasonic treatment (UT) processes.

Alloys and Purity	wt % and Compositions of Al–MgAl_2_O_4_ Master	Master Adding Modes	wt % of MgAl_2_O_4_	*T* (melt, UT) and *t* (Master)	*t* (UT)	Mean Grain Size of MgAl_2_O_4_ in μm	Refinement Degree of Alloy Grain Size	Mold and Size in mm	Melting
Al; C-grade	3 wt % Al– 2Mg–5SiO_2_ with UT [13]	vortex	0.15	720 °C; 20 min	0	0.64	2–3-fold reduction	cast iron mold; Φ 50 × 100; preheated at 500 °C [13]; 300 °C [12]	pit type resistance furnace; clay graphite crucible
					30 s		11–12-fold reduction		
Al–1Mg; C-grade	Al–1Mg– 0.1SiO_2_ [12]	vortex	0.055	750 °C; 30 min	0	–	~2-fold reduction		
			0.085		5 min	~0.62	7–8-fold reduction		
Al–4Mg; C-grade	Al–1Mg– 2H_3_BO_3_ [7]	simple	0	750 °C; 15 min	5 min	none	~1.1-fold reduction	Φ 20 × 80; preheated	resistance furnace; graphite crucible
			0.58		0	~2	3–4-fold reduction;		
					5 min	~0.70	7–8-fold reduction		
Al–4Mg; C-grade	Al–1Mg– 5.2H_3_BO_3_ [18]	simple	3	750 °C; 15 min	0	~3	3–4-fold reduction	cast iron mold; Φ 20 × 120; preheated	resistance furnace; graphite crucible
					5 min	~0.35	7–8-fold reduction		

C-grade: commercial grade purity of alloys; *T* (melt, UT): melt heating temperature after the addition of the master alloys and UT temperature; *t* (Master): melt holding time after the addition of the master alloys; *t* (UT): UT time.

**Table 2:** The theoretical lattice misfits (*f*) for the parallel crystal planes between Al and MgAl_2_O_4_ in Figure 3a.

Al/ MgAl_2_O_4_	*f* (%)		
	{400}	$\left\{2\bar{2}0\right\}$	{040}
{200}	0.20		
2*d-*{2$\bar{2}$0}		0.20	
{020}			0.20
